# FIRST STEPS – a randomized controlled trial on the evaluation of the implementation and effectiveness of two early prevention programs for promoting the social integration and a healthy development of children with an immigrant background from 0–3

**DOI:** 10.1186/s40359-015-0078-z

**Published:** 2015-07-03

**Authors:** Judith Lebiger-Vogel, Constanze Rickmeyer, Annette Busse, Korinna Fritzemeyer, Bernhard Rüger, Marianne Leuzinger-Bohleber

**Affiliations:** Sigmund-Freud-Institut, Frankfurt/Main, Germany, University of Kassel, Kassel, Germany; Institute for Statistics, Maximilian University of Munich, Munich, Germany

**Keywords:** Psychoanalysis, Early prevention, Immigrant background, Migration, Integration, Parent–child-relationship, Early parenting, Attachment, Child development

## Abstract

**Background:**

The social integration of children with an immigrant background has become one of the most urgent social responsibilities in Germany. They are more likely to live in high-risk environments and are disadvantaged with respect to health related variables as well as educationally. Quite a number of projects supporting their integration into the German society exist although many are hardly scientifically evaluated. Most of them focus on the acquisition of German language and therefore address older children (and adults). However, international experts agree that social integration is not only a matter of language but also of earlier developmental processes of children in their first months of life connected to adequate early parenting.

**Methods/Design:**

The model project FIRST STEPS focuses on earliest prevention for children with an immigrant background, supporting their parents in the critical phase of migration and early parenthood. In a prospective randomized comparison group design the effectiveness of a psychoanalytically oriented early prevention program (intervention A) is compared to the outcomes of groups offered by paraprofessionals with an immigrant background (intervention B). Intervention A is a professional offer supporting immigrant families based on developmental psychological and on knowledge on early parenting. 180 families are randomly assigned to intervention A or B. They are supported during the first 3 years of the children’s lives. Social and family stressors, the quality of the parent–child-interaction, child attachment security, the affective, cognitive and social-emotional development of the children and the social integration of the families are assessed during and after the intervention.

**Discussion:**

The project aims at evaluating the implementation as well as the short- and long-term effectiveness of psychoanalytically oriented intervention A compared to the outcomes of intervention B. It is expected that professionally supported early parenting (intervention A) improves the social-emotional, cognitive and language development of immigrant children as well as the social integration of their families to a greater extent than in the comparison groups. In case the model project proves to be effective, a rollout across Germany is possible. Due to the “difficult-to-reach” immigrant families challenges in recruitment, uptake and retention of participants are anticipated.

**Trial registration:**

DRKS-ID: DRKS00004632, trial registration date: 05.02.2013

## Background

Children with an immigrant background are still disadvantaged concerning health related variables such as obesity or psychopathological problems (Kurth & Schaffrath Rosario 2007; Hölling et al. 2008; Rattay et al. 2012; https://www.bmas.de/SharedDocs/Downloads/DE/PDF-Publikationen-DinA4/a334-4-armuts-reichtumsbericht-2013.pdf?__blob=publicationFile; Autorengruppe Bildungsberichterstattung 2012), their educational achievement and are more likely to live in high-risk environments (Sachverständigenrat deutscher Stiftungen für Integration und Migration (SVR) GmbH: Deutschlands Wandel zum modernen Einwanderungsland. Jahresgutachten 2014; Leuzinger-Bohleber et al. 2006; Leuzinger-Bohleber et al. 2009; Leuzinger-Bohleber et al. 2011).^1^ However, it is not the immigrant background per se that puts these children at risk of becoming disadvantaged but it is rather the factors that are associated with their parents´ migration, psychological factors due to the different phases of migration and socio-economic factors (low socio-economic status, unemployment, insecure institutional status etc.) that create difficult developmental environments for these children. Children of mothers who have not lived in Germany for a long time are particularly disadvantaged, because the mothers are in an emotionally insecure situation themselves. They have to get along in a new surrounding while not having experienced attachment figures – such as their own parents and siblings – at reach for support. Especially during the vulnerable time after the birth of a child the mothers often feel alone and isolated and the risks of social withdrawal, isolation and depression are significant. The mother’s stress can have a negative impact on the emotional quality of the early mother-child relationship and attachment security and thus brings about an additional disadvantage for the children, placing them in a special risk group among the immigrant population. Results of the Frankfurt prevention study (Leuzinger-Bohleber et al. 2006; Leuzinger-Bohleber et al. 2009; Leuzinger-Bohleber et al. 2011) indicated that a poor or lacking early integration can be associated with a potential disruption of “natural attachment” in the course of migration and young motherhood.

In addition we know that if children – closely attached to their primary caregivers – unconsciously notice that their parents, especially their mother, suffer from severe homesickness or have not really “arrived” in the country of immigration emotionally, they will perceive turning to the culture and language of the immigration country as a betrayal and a turning away from their parents or mother. Often this kind of loyalty conflict keeps children with an immigrant background from successfully learning the language of the immigration country and integrating psychosocially (King 2007; Leuzinger-Bohleber 2009). Unconsciously they identify with their parents’ losses possibly leading to behavioural difficulties, school failure and/or depression if not processed thoroughly.

Especially findings from attachment research regarding disorganized attached children (Type D) are alarming. As known from longitudinal studies, children who show this kind of attachment style have the worst prognosis and will show aggressive-destructive behaviour, worse school achievement and massive psychological problems already during elementary school in all probability if they do not receive early help and support (Lyons-Ruth et al. 1993; Solomon et al. 1995; Green and Goldwyn 2002; Moss et al. 2004; Cassidy and Shaver 2008; Stacks and Oshio 2009). A notable number of these children come from families with an immigrant background, who due to factors which are often associated with migration such as experiences of violence in their home country, social isolation after marriage migration and resp. low social-economic status are severely stressed or even traumatized (Batista-Pinto Wiese 2010; Fazel et al. 2005; Leuzinger-Bohleber 2012; Schechter and Rusconi Serpa 2014).

Quite a number of projects, which promote the social integration of children with an immigrant background exist in Germany. Most of them focus on acquiring the German language and thus address older children or adults (Friedrich and Siegert 2009; Lösel et al. 2006) and do not explicitly take into account the children’s well-being. Infants however, only begin to actively acquire language during their second year of life and during infancy learning their mother’s language is most important. It is well known that the development of language is based on early “embodied” and preverbal relationships from the beginning on. Stern (1985) studied the different stages of the development of the self and showed that the “verbal self” in the second year of life is built upon the earlier stages of this development (the emergent self, the core self etc.). Thus integration approaches, which solely focus on acquiring the German language, are limited to this aspect and are not effective for children between the age of zero and three (infants and toddlers).

Some of the already existing projects supporting children with an immigrant background have been evaluated. The quality of these evaluations can however often be regarded as problematic and the effectiveness of programs conducted by lay helpers with an immigrant background has often not been thoroughly explored (Friedrich and Siegert 2009; Lösel et al. 2006). In addition many projects deploy lay helpers although the outcome of early prevention projects seems to be positively influenced by the professionalism of staff (Olds et al. 2008; Holodynski et al. 2007). Systematic integration projects with the goal to optimize the first environmental and relationship experiences of children with an immigrant background are scarcely evident in Germany. However research findings strongly suggest such an approach, because infants, who grow up in a positive and emotionally secure environment are more creative, show less aggression, show better cognitive, affective and social-emotional development and learn languages more easily (Aviezer et al. 2002; DeKlyen and Greenberg 2008; Fearon et al. 2010; Sroufe et al. 2005; Thompson 2008; Van IJzendoorn et al. 1995). Therefore one can assume that approaches which take early relationships within the direct living environment as a starting point (during this age particularly the core family), could also improve integration of children with an immigrant background during infancy and toddler age. Furthermore, language acquisition as well as the development of communicative, social competencies cannot fully be understood if they are not viewed within the context of early relationship experiences (Emde and Leuzinger-Bohleber 2014; Korntheuer et al. 2010).

FIRST STEPS is a psychoanalytically oriented prevention program for immigrant families offered from the time of pregnancy until entering kindergarten. It focuses on the specific challenges and needs of families with an immigrant background and seeks to optimize the early developmental environment of children at risk of growing up disadvantaged due to their parents’ acute migration (and possible stress).

The project was conceptualized in the context of the IDeA^2^ Center and is implemented by the Sigmund-Freud-Institut (SFI; Frankfurt/Main) in collaboration with the Anna-Freud-Institut (Frankfurt/Main). IDeA represents a large, interdisciplinary research center, a cooperation between the Goethe University Frankfurt, the German Institute for International Educational Research (DIPF) and the SFI.^3^ It was established within the framework of the LOEWE^4^ initiative, a large promotional programme fostering excellent research in the federal state of Hessen, Germany. FIRST STEPS serves as a scientifically evaluated model project in the sense that its success would enable the implementation in other communities and cities in Germany, independently from regional specificities such as the ethnic composition of the immigration population, project staff and context of recruitment. In a second step it is planned to also implement FIRST STEPS in Berlin. This offers the opportunity of testing the workability and practicality of dissemination, recruitment strategies and possible needs for adaption of FIRST STEPS in another institutional context.

### Goals of FIRST STEPS

FIRST STEPS seeks to contribute to early prevention and improving the social integration as well as a healthy development of children coming from social marginal groups of society, which are hard-to-reach (see below). In supporting mothers with an immigrant background and in promoting infant-mother (and father) relationships from its earliest point on – pregnancy – we hope that FIRST STEPS will facilitate successful parent–child interaction from the time the natural window for its development opens. In doing so, the project intends to promote attachment security and a positive child development. As empirical attachment, neurobiological and epigenetic research show, attachment security is a protective factor for cognitive, socio-emotional and language development as well as academic success (Berlin et al. 2008). Furthermore, it is expected that by promoting parents’ reflective functioning (Fonagy et al. 2002), adequate emotion regulation, processing of losses caused by migration, parenting behaviour (e.g. responding to their infant’s cues consistently) as well as cultural competencies parents’ and children’s psychosocial integration will be facilitated. Negative primary (e.g. poorly educated parents) and secondary family background related effects on the child (Boudon 1974, e.g. parental educational decisions when poorly informed about or afraid of the educational system) would be weakened by this kind of support.

In a prospective randomized comparison group design the project aims at evaluating the implementation as well as the short- and long-term effectiveness of FIRST STEPS (intervention A) in comparison to an intervention being offered by paraprofessionals (intervention B). A variety of instruments are applied at different times of measurement during the intervention as well as afterwards when the children attend kindergarten (follow-up; see below).

## Methods/Design

### Participants, inclusion and exclusion criteria

FIRST STEPS addresses pregnant first-generation immigrant women (from the second trimester on, and their husbands) who have no or little knowledge of the German language and have not been living in Germany for longer than 3 years (in accordance with the population of the integration courses, see Rother N, http://www.bamf.de/SharedDocs/Anlagen/DE/Publikationen/WorkingPapers/wp19-Integrationspanel.pdf?__blob=publicationFile). Most of our participants have a low socio-economic status and are hard-to-reach. In this context “hard-to-reach” means the participants often fail to access social and community services (e.g. family support services) and therefore remain unprovided (Doherty et al. 2004). It is difficult to recruit this population and a lot of effort is needed to get the participants to commit to the prevention program and to stay involved over time (Cooney et al. 2007).

### Recruitment

Participants are recruited in obligatory integration courses (on German language and "culture") at three different regional non-profit social service centres in Frankfurt/Main. These courses were implemented nationwide in 2004 and are part of the political reactions to the clarion call of the early PISA studies and the late realization that Germany has become an immigration country but had mainly missed out on socially integrating its immigrants.

### Interventions/observational groups

The FIRST STEPS study compares two prevention programs which are both offered from the time of pregnancy until entering kindergarten: A broader, more individual and complex psychoanalytically oriented intervention, the FIRST STEPS intervention (A) and a a less complex standard intervention being offered by paraprofessionals (B).

#### Intervention A - FIRST STEPS

The psychoanalytically trained FIRST STEPS project staff, mostly mothers with an immigrant background themselves, support the women (and their husbands) ideally already during pregnancy. Hence they build an emotional relationship with them in order to continue their support after the birth of the child. This support should help the women to avoid withdrawal into isolation. Afterwards the project staffs continue to accompany and support mothers and children both in group contacts (moderated weekly groups with two project staff members) and individual contacts (via telephone, home visits) until the children enter kindergarten at the age of around three. The FIRST STEPS approach is curriculum-based. The training of the project staff includes psychoanalytic case-supervision, practice reflection with the coordinator of the practical implementation^5^ as well as regular supervision with child and adolescent psychotherapists. The manualised curriculum, which has been developed by the coordinator of the practical implementation, is based on psychoanalytic and empirical developmental psychology and will be published in the near feature. The conceptualisations closely refer to other already evaluated psychoanalytically oriented parenting programs (Emde and Robinson 2000; Meurs et al. 2006; Parens et al. 1995). The training sensitizes staff for the processes of transference and counter-transference allowing for a deeper understanding of the women’s situation and children’s needs. The project staffs learn to develop a psychoanalytical “mind-set” and to create a holding and containing function (Bion 1963) in contact with the individual women as well as during the group sessions. Thus they can serve as role models and as a “secure base” (Bowlby 1969, p. 325) for the mothers, supporting them in the vulnerable phase of their early motherhood. Their consultation focuses on individual needs of the mothers and children as well as questions and concerns the families might have concerning the child’s development. The project staff thereby supports parenting competences (e.g. reflective functioning, adequate emotion regulation). Furthermore, questions concerning migration problems of the families (supporting them to consult institutions, clinics, social and mental health care services, language courses, educational institutions etc.) are addressed. Coping with losses associated with the women’s and families’ migration is supported.

#### Intervention B

The mothers and children in intervention B take part in self-organized open “mother-child groups” led by female paraprofessionals with an immigrant background^6^, who are mostly mothers themselves, pass on their experiences to the mothers and invite for social exchange. The paraprofessionals are only instructed and informed about the study very basically, including the aimed duration of the intervention until children enter kindergarten and research instruments, and are free to conduct and organize their groups according to their views and their experience as immigrants and mothers. They do not receive any support with regard to contents and have no contact with the project organization and implementation other than with the research team collecting data about mothers and children.

### Assessments

A variety of instruments are applied at five different times of measurement; four in the course of the intervention, one directly afterwards, when the children enter kindergarten (t2: 2,5-5 months, t3: 13–15 months, t4: 24–27 months, t5: 36–39 months) and one time of measurement after the intervention, when the children attend kindergarten (t6: 8–14 months after kindergarten entrance).

Sociodemographic information of the families (baseline assessment, t1) is assessed at the beginning of the intervention by using a self-report questionnaire, which was developed by the Bundesamt für Migration und Flüchtlinge (BAMF, German Federal Office for Migration and Refugees).^7^ The BAMF questionnaire is complemented by an additional set of questions concerning the mother’s integration called the Hertie Belonging Scale, a scale developed by the research team. Furthermore, the project staff evaluates family stressors and social support of the families during the course of the intervention by using the Heidelberger Belastungsskala (HBS-L) (Stasch 2009), a standardized screening scale. In addition the mothers’ subjective daily stressors and life-satisfaction are assessed twice (t4 and t5) using a self-report questionnaire, the Everyday Stressors Index (ESI, Jäkel and Leyendecker 2008). Mothers’ depressive symptoms are assessed after birth (t2) with a self-report questionnaire, which is used as a screening instrument for post-partum depression. The mothers are asked to fill in the ADS Scale (Allgemeine Depressionsskala, Hautzinger and Bailer 2002) with the help of the project staff. The cognitive and motor development of the children are assessed by trained psychologists at age 2 (t4) using the German version of the Bayley Scales of Infant Development II (Reuner et al. 2007). Furthermore, the emotional quality of mother-child interaction is assessed at four different times of measurement (t2, t3, t4 and t5). Therefore videotaped mother-child interactions are blindly rated by independent and trained psychologists with the help of the Emotional Availability Scales (Biringen Z, http://emotionalavailability.com), an observational instrument with a dyadic focus.

After the intervention, when the children enter kindergarten (t5) an Integration Questionnaire (including the Hertie Belonging Scale) is applied in order to gain information on the mothers’ successful participation resp. continuation of the integration/language courses and the mothers’ integration. Furthermore, the mothers’ satisfaction with the intervention is assessed with the help of a half standardized questionnaire in form of an interview.

**Fig. 1:**
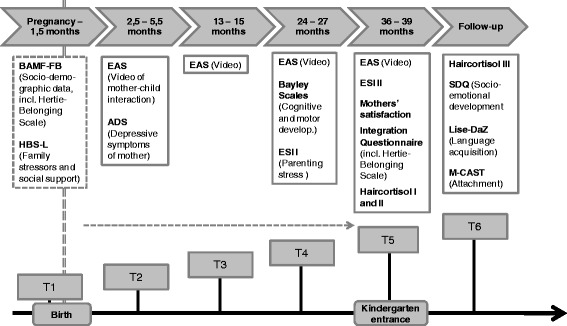
Timeline of assessed variables and times of measurements. Legend: BAMF-FB: Questionnaire from the Federal Office for Migration and Refugees; Hertie-Belonging Scale: half standardized rating form – information on the mothers’ integration; HBS-L: Heidelberger Belastungsskala (scale on family stressors and social support); EAS: Emotional Availability Scales; Bayley: Bayley Scales of Infant Development II; ADS: Center for Epidemiologic Studies-Depression Scale; ESI: Everyday Stressors Index; Integration Questionnaire: Half standardized rating form – information on the mothers’ successful attendance of the integration course and integration (including selected BAMF items and the Hertie Belonging Scale); SDQ: Strengths and Difficulties Questionnaire; LiSe-DaZ: Linguistic Language Development Survey – German as Second Language; MCAST: Machester Child Attachment Story Task

In addition, the children’s stress level, socio-emotional as well as language development is assessed during follow-up. Thereby the children’s hair cortisol level is measured as a marker of stress when the children enter kindergarten (t5), 6 weeks afterwards as well as 1 year later (t6). Furthermore, the kindergarten teachers evaluate the children’s behaviour (aggressiveness, hyperactivity, anxiety and social competence) using the Strengths and Difficulties Questionnaire (SDQ, Becker et al. 2004a; Klasen et al. 2000; Woerner et al. 2002; Woerner et al. 2004) a year after entering kindergarten (t6). Also at t6 the children’s attachment security is assessed by trained and independent psychologists with the help of the Manchester Child Attachment Story Task (MCAST) (Green et al. 2000). At about the same time (t6) the children’s German language development is assessed by trained linguists with the help of the LiSe-DaZ (Linguistic Language Development Survey – German as Second Language, Schulz and Tracy 2011). This is a standardized language test, which is conceptualized and standardized for children who learn German as a second language. The assessed variables and times of measurement are presented in Fig. 1.

### Objectives and hypotheses

The purpose of the trial is to address the following issues:

- Differential short and long-term efficacy of two prevention programs (interventions A & B) for children with an immigrant background and their mothers and
- Course and stability of prevention effects.

The hypotheses assume that FIRST STEPS (intervention A), which individually addresses the families´ particular needs, is more effective compared to the intervention being offered by paraprofessionals (intervention B) concerning the following outcomes.

The first set of hypotheses assumes that it is more effective in terms of the children’s development.

#### Hypotheses on primary outcome

The primary outcomes will be measured shortly after entering (t5) and after 1 year of kindergarten (t6).

*Hypothesis 1:* When entering kindergarten (t5) and after 1 year in kindergarten (t6) the children in intervention A show lower levels of stress compared to the children in intervention B (cortisol level measured by hair cortisol).

*Hypothesis 2:* After 1 year in kindergarten (t6) the children in intervention A show a better language development compared to the children in intervention B (measured by the Linguistic Language Development Survey – German as Second Language, LiSe-DaZ).

*Hypothesis 3:* After 1 year in kindergarten (t6) the children in intervention A show a better socio-emotional development compared to the children in intervention B (measured by the Strengths and Difficulties Questionnaire, SDQ).

#### Hypotheses on secondary outcome

*Hypothesis 4:* At time of measurement t4 the children in intervention A show a better cognitive and motor development compared to the children in intervention B (measured by Bayley Scales of Infant Development II).

*Hypothesis 5:* At time of measurement t5 the children in intervention A show a better relationship to their primary caregiver (mother) compared to the children in intervention B (indicator: parent–child-interaction measured by the Emotional Availability Scales, EAS).

*Hypothesis 6:* The children in intervention A show after 1 year in kindergarten (t6) more often a secure attachment style (Type B) compared to the children in intervention B (measured by the Manchester Child Attachment Story Task, MCAST).

The second set of hypotheses assumes that intervention A is more effective in terms of the mothers’ psychosocial integration and language development than intervention B.

#### Hypotheses on primary outcome

*Hypothesis 7:* At time of measurement t5 more mothers in intervention A successfully have completed the integration course in comparison to those in intervention B (measured by the Integration Questionnaire with repeated assessment of questions of the BAMF questionnaire)

*Hypothesis 8:* At time of measurement t5 the mothers in intervention A are socially more oriented towards the host country, in comparison to those in intervention B (measured with the Hertie Belonging Scale).

#### Hypotheses on secondary outcome

*Hypothesis 9:* At time of measurement t5 the mothers in intervention A show a higher satisfaction with the intervention in comparison to the mothers who took part in intervention B.

### Design

**Fig. 2:**
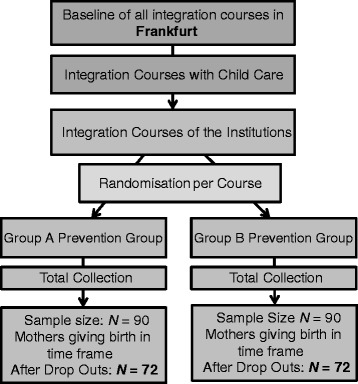
Research design

Figure 2 summarizes the study design. The individual participants (children and their parents) were not aware of their group assignment, that is, if they were participating in intervention A (FIRST STEPS) or in intervention B. The integration courses which the women are attending are randomly assigned to the two different interventions A and B, because women from the same integration course cannot be referred to different interventions (cluster-randomization).

### Outcome

#### Primary outcome

1. One primary outcome criterion is the *stress level of the children* measured by the children’s hair cortisol level a week before kindergarten entrance, 6 weeks afterwards as well as 1 year afterwards. Measuring the concentration of cortisol in hair is a new and promising method to measure cortisol (Stadler and Kirschbaum 2012). For many years cortisol was obtained primarily from blood plasma or urine, whereas later approaches added saliva and feces for non-invasive monitoring of HPA functioning. These methods are limited in the temporal range of assessment and are only snapshots of HPA activity. However being incorporated into the growing hair, hair cortisol concentrations are assumed to provide a retrospective reflection of integrated cortisol secretion over periods of several months (Stadler and Kirschbaum 2012). Over the past years supportive evidence has accumulated regarding several fundamental characteristics of hair cortisol concentrations, including its validity as an index of long-term systemic cortisol levels both in animals (Fairbanks et al. 2011) and in human participants (Manenschijn et al. 2011), its reliability across repeated assessments (Stadler et al. 2012) and its relative robustness to a range of potential confounding influences (Stadler and Kirschbaum 2012).
2. Another primary outcome criterion is the *children’s knowledge of the German language*, assessed a year after kindergarten entrance by a trained linguist with the help of the LiSe-DaZ (Linguistische Sprachstandsdiagnostik - Deutsch als Zweitsprache, Schulz and Tracy 2011), a standardized language test. Language acquisition is critical for the children’s later school success (Gantefort and Roth 2010; Niklas et al. 2011). The LiSeDaZ enables to measure children’s development in core grammatical areas and offers separate norms for multilingual children, which take into account the age of first exposure and the length of exposure to the German language (Schulz and Tracy 2011).
3. A third primary outcome criterion is the *children’s socio-emotional development*, evaluated at kindergarten age by kindergarten teachers using the Strengths and Difficulties Questionnaire (SDQ, Becker et al. 2004a). With the help of the SDQ the following subscales can be assessed: prosocial behavior, hyperactivity, emotional problems and conduct problems with peers. The SDQ is not only a practical and economical, but also a valid and reliable questionnaire for use in the framework of a multi-dimensional behavioural assessment, and appears to be well suited for screening purposes, longitudinal monitoring of therapeutic effects, and scientific research purposes (Becker et al. 2004b; Klasen et al. 2000; Woerner et al. 2002).
4. A fourth primary outcome criteria on the part of the mothers is the *mother’s integration* (among others mothers’ successful completion of integration courses, employment/occupational comeback, interest in host country), measured by the repeated assessment of a part of the BAMF questionnaire and the Hertie Belonging Scale at the end of the intervention as indicators of successful integration.

#### Secondary outcomes

The secondary outcomes are:

1. *The children’s cognitive and motor development*, assessed by trained psychologists at age 2 (t4) using the German version of the Bayley Scales of Infant Development II (BSID, Reuner et al. 2007). The BSID II evaluates infants along three scales: a cognitive, a motor and a behaviour scale. The scales have been used extensively worldwide to assess the development of infants and are known to have high reliability and validity (Vohr et al. 2012).
2. *The emotional quality of the of mother-child interaction*, assessed at four different times of measurement (t2 to t5) by independent and trained psychologist using the Emotional Availability Scales (EAS, http://emotionalavailability.com). The EAS allow for a detailed look at caregiver-child interactions by rating the dyad on six dimensions, four focusing on the parent’s behaviour (sensitivity, structuring, non-intrusiveness, non-hostility) and two focusing on the child’s behaviour (child responsiveness, child involvement of the caregiver). Significant findings have been reported about the positive relationship between parental EA and children’s attachment security (Biringen 2000) and there is a large body of empirical research using the EA Scales (Biringen et al. 2014). It is of particular relevance that the EAS dimensions can be rated independently of the caregivers’ cultural background (Biringen 2009; Biringen et al. 2014). Ziv et al. (2000) for example reported cross-cultural applicability of the EAS by examining links with attachment. In addition the EAS are an instrument that is sensitive to change related to a program of intervention (for an overview see Biringen et al. 2014).
3. *The children’s attachment style*, assessed by trained psychologists using the MCAST (Green et al. 2000) 1 year after entering kindergarten (t6). The MCAST, a narrative story stem task that involves playing with dolls, is a validated, structured measure that evaluates young children’s attachment representations through the use of play scenarios allowing for differentiation between four overall attachment classifications: secure attachment, insecure-ambivalent attachment, insecure-avoidant attachment und insecure-disorganized attachment representations.
4. *The mothers’ satisfaction with the intervention,* assessed after the intervention by using a half-standardised questionnaire in form of a resumeé interview at t5.

### Sample size calculation - power analysis

The sample size calculation resp. power analysis is based on *α* = 0.05 at a power of 0.80, using pilot data concerning the number of participants in the language courses. Concerning the different primary outcomes (children’s stress level, language development, socio-emotional development and mothers’ integration) we expect effect sizes of at least *d* = 0.5. Then the sample size for analysis of variance in a RCT study would be *n* = 63. But we applied a CRCT study. Therefore we have to correct the *n* in a corrected n* according to the following formula introduced by Eldridge et al. (2006), providing a conservative estimate of sample size requirements for trials using cluster-level analyses weighted by cluster size:

$$ n* = \left\{\ 1 + \left[\ \left(1+CV{}^2\right)\ \mathrm{x}\ \mathrm{m}\ \hbox{--}\ 1\ \right]\ \mathrm{x}ICC\right\}\ \mathrm{x}\ n $$

The formula consists of the coefficient of variation *CV* for trials with unequal cluster sizes (that means with unequal sizes of language courses) and the intraclass-correlation coefficient *ICC* within the clusters and the mean cluster size *m*. Using the findings of a pilot study, the estimated coefficient of variation is *CV* = 0.4 (rather smaller) and the estimated intra class correlation coefficient (based on the pre-post differences including the *CV*) *ICC* = 0.1 (rather smaller). Furthermore, we expect a mean cluster size of *m* = 2 pregnant women in each course (cluster).

The corrected sample size would therefore be n* = 1.132 × 63 = 71.32. Thus the minimum sample size would be 72 pregnant women per treatment and resp. 36 integration courses should be selected per treatment. However under consideration of a 20 % drop-out rate the corrected minimum sample size is n* = n* × 1.25 = 90 women per treatment and thus a number of 45 courses per treatment should be selected (see Fig. 2).

### Representativeness of the sample and sample selection (at cluster level)

To control for internal representativeness homogeneity tests are applied in order to verify that the two treatment groups are homogenous and in order to test for potential bias due to drop-outs or missing values. To prove that our sample is similar to the German population of immigrants that take part in integration courses nationwide (control of external representativeness) the BAMF questionnaire is applied.

### Randomization

As Fig. 2 shows, randomization was performed at cluster level (integration course). A cluster-randomization was applied instead of a single randomization, because women from the same integration course should be assigned to the same intervention and not to different interventions. The statisticians, uninformed about the identity of the integration courses, used a table of random numbers for randomizing the 90 integration courses included in the study. Individual participants (children and their parents) were not aware of their group assignment, that is, if they were participating in the psychoanalytical intervention or the intervention provided by paraprofessionals.

### Statistical analysis

The design is an analysis of variance design with repeated measures. The main instruments for analysis applied are Analysis of variance (ANOVA) as well as Analysis of covariance (ANCOVA) models. The main factor is the intervention group (A or B) and the most relevant secondary factor is the initial value (baseline value). All baseline characteristics will be described at the individual level. Relevant characteristics will be added if applicable as covariates to the models. Six main assessments encompass the treatment phase and follow-up.

### Ethical issues

The Ethic Review Commission of the Federal Chamber of Psychotherapists of the State of Hessen, Germany, has approved the final study protocol and the final version of the written informed consent form. Written consent was obtained from each participating family. The trial will be carried out in keeping with local legal and regulatory requirements.

## Discussion

This trial has one major goal: to compare the effectiveness of two different interventions on the development of children with an immigrant background in Germany and the integration of their mothers. Based on empirical findings it is expected that professionally supported good early relationship experience improves long-term integration of immigrant children. In a prospective randomized comparison group design the project aims at evaluating the implementation as well as the short- and long-term effectiveness of FIRST STEPS (intervention A) in comparison to an intervention being offered by paraprofessionals (intervention B). Anticipated is that supporting the earliest parent-child-interactions and parenting capacities in a professional psychoanalytically oriented intervention (A) will have a greater positive impact on the mothers’ integration, on children’s affective, socio-emotional and cognitive development and on the quality of the parent-child relationship than the intervention provided by paraprofessionals with an immigrant background in the more open “mother-child groups” (B). We tried to reach comparable doses of intervention in the course of the 3 years of intervention for ensuring that effects would be caused by the type of intervention and would not just be a matter of more or less intervention.

In any case, number and timing of group sessions and individual contacts will be documented. In this trial, a high quality is assured by an independent assessment. Due to the high expected attrition rate in this population at risk, drop-out is thoroughly documented and respective analyses are being planned.

As mentioned before, high quality evaluation of prevention projects for young children with an immigrant background and their families have been scarce and to our knowledge an RCT comparison group design is non-existent in Germany. Thus the study has the status of a model project. If the prevention offers prove to be effective and sustaining, the empirically based prevention programs may be implemented in other German cities and migrant populations as well. Thus the study will prove to have a practical and political relevance.

## Abbreviations

ADS: Allgemeine Depressionsskala
ANCOVA: Analysis of covariance
ANOVA: Analysis of variance
BAMF: Bundesamt für Migration und Flüchtlinge (German Federal Office for Migration and Refugees)
BSID: Bayley Scales of Infant Development
DIPF: Deutsches Institut für internationale pädagogische Forschung (German Institute for International Educational Research)
EAS: Emotional Availability Scales
ESI: Everyday Stressors Index
HBS-L: Heidelberger Belastungsskala (scale on family stressors and social support)
IDeA: Individual Development and Adaptive Education of Children at Risk
LiSe-DaZ: Linguistische Sprachstandserhebung – Deutsch als Zweitsprache (Linguistic Language Development Survey – German as Second Language)
LOEWE: Landesoffensive zur Entwicklung wissenschaftlich-ökonomischer Exzellenz (Political strategy of the State of Hessen for strengthening the development of scientific and economic excellence)
MCAST: Manchester Child Attachment Story Task
SDQ: Strengths and Difficulties Questionnaire
SFI: Sigmund-Freud-Institut

## Footnotes

1 In 2010 48 % of children living in families with a migrant background grew up exposed to at least one situation of risk such as unemployed, low-income or educationally disadvantaged parents.

2 IDEA = Individual Development and Adaptive Education of Children at Risk; http://www.idea-frankfurt.eu/de.

3 At this point about 120 researchers from different disciplines such as pedagogics, psychology, psychoanalysis, neurosciences and different subject didactics work together at the center. Around 50 different research projects are realized.

4 **L**andes-**O**ffensive zur **E**ntwicklung **W**issenschaftlich-ökonomischer-**E**xzellenz (Political strategy of the State of Hessen for strengthening the development of scientific and economic excellence)

5 Child and youth-psychoanalyst Claudia Burkhardt-Mußmann (in Frankfurt) and Rose Alheim (in Berlin)

6 Self-organized open mother-child groups have become a common form of similar early prevention projects in Germany (Friedrich & Siegert 2009).

7 This survey gives a broad overview over the personal background of the participants, the already acquired languages and especially German language skills, job and educational issues and their life in Germany. It has been used in a large scale study for the population of the integration courses in Germany.
